# Home parenteral nutrition with an omega-3-fatty-acid-enriched MCT/LCT lipid emulsion in patients with chronic intestinal failure (the HOME study): study protocol for a randomized, controlled, multicenter, international clinical trial

**DOI:** 10.1186/s13063-019-3994-z

**Published:** 2019-12-30

**Authors:** Stanislaw Klek, Cécile Chambrier, Sheldon C. Cooper, Simon Gabe, Marek Kunecki, Loris Pironi, Farooq Rahman, Jacek Sobocki, Kinga Szczepanek, Geert Wanten, Nicole Lincke, Bernhard Glotzbach, Alastair Forbes

**Affiliations:** 1 Stanley Dudrick’s Memorial Hospital, General and Oncology Surgery Unit, 15 Tyniecka Street, 32-050 Skawina, Poland; 2 Hospices Civils de Lyon, Centre Hospitalier Lyon Sud, 165 Chemin du Grand Revoyet, 69495 Pierre-Benite, France; 3 GI Medicine – University Hospitals Birmingham NHS Foundation Trust, Queen Elizabeth Hospital, Mindelsohn Way, Birmingham, B15 2TH UK; 4 St Mark’s Hospital, Northwick Park, Watford Road, Harrow, HA1 3UJ UK; 5 Wojewódzki Specjalistyczny Szpital im. M. Pirogowa w Łodzi, Oddział Chirurgii Ogólnej i Naczyniowej, ul. Wólczańska 191/195, 90-531 Łódź, Poland; 6 Department of Medical and Surgical Science, University of Bologna, St. Orsola-Malpighi Hospital, Via Massarenti, 9, 40138 Bologna, Italy; 7 University College Hospital, 250 Euston Road, London, NW1 2PG UK; 8 Samodzielny Publiczny Szpital Kliniczny im. Prof. dr W. Orlowskiego, Oddzial Kliniczny Zywienia i Chirurgii, ul. Czerniakowska 231, 00-416 Warszawa, Poland; 9 Radboud Universitair Medisch Centrum, Afdeling Maag-, Darm- en Leverziekten, Postbus 9101, 6500 HB Nijmegen, The Netherlands; 10 Medical Scientific Affairs, B. Braun Melsungen AG, Carl-Braun-Str. 1, 34212 Melsungen, Germany; 11 Faculty of Medicine and Health Sciences, University of East Anglia, Norwich, NR4 7TJ UK

**Keywords:** Home parenteral nutrition, Intravenous lipid emulsion, Omega-3 PUFA, Chronic intestinal failure, Liver function, Randomized controlled trial

## Abstract

**Background:**

Home parenteral nutrition (HPN) is a life-preserving therapy for patients with chronic intestinal failure (CIF) indicated for patients who cannot achieve their nutritional requirements by enteral intake. Intravenously administered lipid emulsions (ILEs) are an essential component of HPN, providing energy and essential fatty acids, but can become a risk factor for intestinal-failure-associated liver disease (IFALD). In HPN patients, major effort is taken in the prevention of IFALD. Novel ILEs containing a proportion of omega-3 polyunsaturated fatty acids (n-3 PUFA) could be of benefit, but the data on the use of n-3 PUFA in HPN patients are still limited.

**Methods/design:**

The HOME study is a prospective, randomized, controlled, double-blind, multicenter, international clinical trial conducted in European hospitals that treat HPN patients. A total of 160 patients (80 per group) will be randomly assigned to receive the n-3 PUFA-enriched medium/long-chain triglyceride (MCT/LCT) ILE (Lipidem/Lipoplus® 200 mg/ml, B. Braun Melsungen AG) or the MCT/LCT ILE (Lipofundin® MCT/LCT/Medialipide® 20%, B. Braun Melsungen AG) for a projected period of 8 weeks. The primary endpoint is the combined change of liver function parameters (total bilirubin, aspartate transaminase and alanine transaminase) from baseline to final visit. Secondary objectives are the further evaluation of the safety and tolerability as well as the efficacy of the ILEs.

**Discussion:**

Currently, there are only very few randomized controlled trials (RCTs) investigating the use of ILEs in HPN, and there are very few data at all on the use of n-3 PUFAs. The working hypothesis is that n-3 PUFA-enriched ILE is safe and well-tolerated especially with regard to liver function in patients requiring HPN. The expected outcome is to provide reliable data to support this thesis thanks to a considerable number of CIF patients, consequently to broaden the present evidence on the use of ILEs in HPN.

**Trial registration:**

ClinicalTrials.gov, ID: NCT03282955. Registered on 14 September 2017.

## Background

### Home parenteral nutrition and chronic intestinal failure

Home parenteral nutrition (HPN) is indicated for patients who cannot meet their nutritional requirements by enteral intake and who are able to receive therapy outside an acute care setting [1]. A reduction of the gut function below the minimum necessary for the absorption of macronutrients and/or water and electrolytes, such that intravenously administered supplementation (IVS) is required to maintain health and/or growth, is defined as intestinal failure [2]. For patients with chronic intestinal failure (CIF) who require IVS for a long period, or for the rest of their life, HPN is currently the primary therapy [1].

The management of parenteral nutrition (PN) supply of patients at home varies among different countries and even between hospitals within the same country, but is always subject to thoroughly organized processes adapted to patients’ needs and preferences. Also, the nutritional and fluid needs of HPN patients are very variable and should be determined on the basis of individual patient assessments [3].

### Intravenously administered lipid emulsions and liver function

Intravenously administered lipid emulsions (ILEs) are a main source of energy and essential fatty acids (EFA), by which they are an essential component of PN and should be provided to almost all patients dependent on HPN [4]. The first ILEs developed were derived from cotton seed oil or soybean oil (SO) which have a high content of the omega-6 polyunsaturated fatty acid (n-6 PUFA) linoleic acid (LA, 18:2n-6). SO-based ILE – commonly called “long-chain triglycerides” (LCT) – in particular in higher doses, are considered to cause several nutritional complications due to the pro-inflammatory metabolites of LA and the content of potentially toxic phytosterols [5, 6]. In order to decrease the amount of n-6 PUFA, physical mixtures, consisting of 50:50 SO and coconut oil (containing mainly medium-chain triglycerides (MCT)), structured-lipid (LCT/MCT) and olive oil (OO), ILE have been developed. Several studies have suggested that this so-called second generation of ILEs may have a lower impact on liver function than pure SO-LCT ILEs [7, 8]. Newer ILEs are more complex and include a proportion of fish oil (FO)/omega-3 (n-3) PUFA, i.e., eicosapentaenoic acid (EPA, C20:5n-3) and docosahexanoic acid (DHA, C22:6n-3). EPA and DHA have been described to possess favorable immunomodulatory and anti-inflammatory properties and their use has been shown to be of benefit in various patient populations [9–14].

In HPN patients a major effort is made towards the prevention of, or if it has already occurred, the treatment of, intestinal-failure-associated liver disease (IFALD). There is no standardized definition, but the term IFALD refers to liver injury caused by several factors relating to CIF, including but not limited to PN. The source and the amount of the lipid component of the PN admixture are under special focus. For long-term administration (> 6 months) SO-based ILE should comprise no more than 1 g lipid/kg body weight (BW) per day to prevent hepatic toxicity, i.e., IFALD [15]. Decreasing the amount of lipid, however, is often not an appropriate strategy, in particular if carbohydrates would need to be provided in excess to achieve the total energy goal. Thus, newer ILEs with a more favorable n-6:n-3 PUFA ratio might be promising options for long-term use.

In the past few years, several studies on FO-based ILEs have been conducted to evaluate their potential benefits in treatment and prevention of IFALD, in particular in pediatric patients [16–18]. For adult patients, data on the use of n-3 PUFA ILEs in HPN are still very limited [2, 19]. Some case reports showing beneficial effects from FO/n-3-enriched ILEs in the prevention or treatment of IFALD [20–22] and case series [23, 24] are available. A systematic literature review published in 2018 (search results until November 2015) identified only three randomized controlled trials (RCTs) investigating the effects of ILE in adult patients, altogether covering 110 patients [25]. Just one of them studied an n-3 PUFA-containing ILE [26]. In this study in 73 patients, lower values of total bilirubin, alanine transaminase (ALT) and aspartate transaminase (AST) were detected after 4 weeks of treatment with a mixed FO-containing ILE compared to SO. A recently published study observed 67 patients for 12 months on HPN and compared four ILEs (LCT *n* = 14, MCT/LCT *n* = 18, OO/LCT *n* = 17, and LCT/MCT/OO/FO *n* = 16) [27]. They showed that all four ILEs were safe and had comparable influence on liver function with a slightly better effect from OO/LCT, however recognizing some limitations in interpretation given disparate initial patient characteristics.

### Study objective

This clinical trial aims to investigate the safety and tolerability of an n-3 PUFA-enriched ILE in comparison to an LCT/MCT ILE for a projected period of 8 weeks with special regard to liver function in stable adult HPN patients.

The working hypothesis is that n-3 PUFA-enriched ILE is safe and well-tolerated especially with regard to liver function in patients requiring HPN. The expected outcome is to provide reliable data to support this thesis thanks to a considerable number of CIF patients, consequently to broaden the present evidence on the use of ILEs in HPN.

## Methods/design

### Study design

The HOME study is a prospective, randomized, controlled, double-blind, international, multicenter, phase IV clinical trial with two parallel groups to be conducted in European hospitals that treat HPN patients. It aims to investigate safety and tolerability of an n-3 PUFA-enriched MCT/LCT ILE in stable adult CIF patients on long-term HPN and to show its non-inferiority compared to an MCT/LCT ILE with regard to liver function. A total of 160 (80 per group) eligible patients will be randomly assigned to receive the n-3 PUFA-enriched MCT/LCT ILE or the MCT/LCT ILE within their PN regimen.

The SPIRIT (Standard Protocol Items: Recommendations for Interventional Trials) Checklist for this study can be found in Additional file 2.

### Setting

The settings of the study are the surgical or gastroenterological outpatient clinics of the participating hospitals, where patients are cared for and monitored; however, the PN regimen including the Investigational Product (IP) is administered to patients at their homes. The processes of HPN supply can differ widely between countries and even between hospitals within the same country, but it is intended to follow the local standard procedures in this multicenter study as far as possible. The recruitment period was initially set at 18 months.

### Trial population and eligibility criteria

Patients will be screened from the existing patient pool of the participating hospitals and must meet the following inclusion criteria to be considered eligible for the trial: informed consent has to be available, male or female patients ≥ 18 years of age with CIF receiving HPN including lipids in whom the parenteral macronutrients have not been changed by more than 10% for at least 6 months, and patients must receive ≥ 3.0 g lipids/kg BW per week.

It was decided to present the dose of lipids is presented as grams per kilogram (g/kg) BW/week as the accepted policy in the designing of the HPN regimen is to reduce PN infusion days to a necessary minimum per week, maintaining the obligatory provision at the same time.

A patient who meets any of the following criteria will be excluded from participation: (1) persistent high total bilirubin values in medical history of the last 6 months (> 40 μmol/l), (2) interruption of PN for longer than four continuous weeks in the preceding 6 months, (3) history of cancer and anti-cancer treatment within the last 2 years, (4) hypersensitivity to egg, fish, peanut or soybean protein or to any of the active substances or excipients, (5) treatment with teduglutide in the past or currently, (6) contraindications to IPs (if available from medical records), which are severe hyperlipidemia including severe hypertriglyceridemia (≥ 1000 mg/dl or 11.4 mmol/l), severe coagulopathy, intrahepatic cholestasis, severe hepatic insufficiency, severe renal insufficiency in the absence of renal replacement therapy, acute thromboembolic events, fat embolism, aggravating hemorrhagic diatheses and metabolic acidosis, (7) general contraindications to PN (if available from medical records) including unstable circulatory status with vital threat (states of collapse and shock), acute phase of cardiac infarction or stroke, unstable metabolic conditions (e.g., decompensated diabetes mellitus, severe sepsis, coma of unknown origin), inadequate cellular oxygen supply, disturbances of the electrolyte and fluid balance (e.g., hypokalemia and hypotonic dehydration), acute pulmonary edema, decompensated cardiac insufficiency, (8) positive test for HIV, hepatitis B or C (from medical history), (9) known or suspected drug or alcohol dependency, (10) patients who are unwilling or mentally and/or physically unable to adhere to study procedures, (11) participation in another interventional clinical trial in parallel or within 3 months prior to the start of this clinical trial, (12) any medical condition that in the opinion of the investigator might put the subject at risk or interfere with patient participation, (13) women of childbearing potential testing positive on standard pregnancy test (urine dipstick), (14) lactating women, (15) women of childbearing potential who do not agree to apply adequate contraception and (16) persons of legal age who are the subject of a legal protection measure or who are unable to express their consent.

### Investigational Products (IPs)

The test IP Lipidem/Lipoplus® 200 mg/ml (B. Braun Melsungen AG, subsequently referred to as Lipidem® 200 mg/ml) is a milky-white oil-in-water emulsion for infusion. One liter of emulsion contains 100.0 g MCT, 80.0 g LCT and 20.0 g n-3 PUFA and provides the following amounts of EFAs: 38.4–46.4 g linoleic acid (n-6 PUFA), 4.0–8.8 g alpha-linolenic acid (n-3 PUFA), and 8.6–17.2 g EPA and DHA in sum. Additional excipients are all-rac-α-tocopherol, egg lecithin, glycerol, ascorbyl palmitate, 2.6 mmol/l sodium as sodium hydroxide and sodium oleat and water for injection. The energy content of 1000 ml Lipidem® 200 mg/ml is 7990 kJ ≈ 1910 kcal, the theoretical osmolality is approximately 410 mOsm/kg and pH range is 6.5–8.5 (NaOH or HCl < 0.5 mmol/l for adjustment to pH 7.4).

The reference IP Lipofundin® MCT/LCT/Medialipide® 20% (100 mg/ml + 100 mg/ml) (B. Braun Melsungen AG, subsequently referred to as Lipofundin® MCT/LCT 20%) is a milky-white oil-in-water emulsion for infusion. One liter contains: 100 g MCT and 100 g LCT and provides the following amounts of EFAs: 48.0–58.0 g linoleic acid (n-6 PUFA) and 5.0–11.0 g alpha-linolenic acid (n-3 PUFA) (n-3 PUFA). Additional ingredients are all-rac-α-tocopherol, egg lecithin, glycerol, sodium oleat and water for injection. The energy content of 1000 ml Lipofundin® MCT/LCT 20% is 8095 kJ ≈ 1935 kcal, the theoretical osmolarity is approximately 380 mOsm/l and pH range is 6.5–8.5 (NaOH or HCl < 0.5 mmol/l for adjustment to pH 7.4).

The administration of the IP is performed intravenously. The IP will be delivered as the lipid part of the PN. Besides the lipid, PN contains glucose, amino acids, electrolytes, trace elements and vitamins and will be administered according to the individual patient’s normal prescription. The weekly dose of IP will be at least 3.0 g lipid/kg BW (corresponding to 15 ml emulsion/kg BW).

### Randomization, blinding and unblinding

Eligible patients will receive a patient number indicating country, study site and randomization number. The randomization to either treatment will be in a 1:1 ratio with stratification for study center. A list of treatment assignments has been generated prior to the initiation of the study using consecutive blocks with the order of assignments chosen at random by an independent biometrician. The study is double-blind.

The processes of HPN supply differ between the study sites. The PN is prepared either in stocks as all-in-one admixtures in the hospital pharmacy or a compounding unit (CU) of a pharmacy service company or it is provided in multi-chamber bags to which the IP is added or it is mixed from the single nutrients on a daily base. In this multicenter clinical trial it is intended to follow the hospitals’ local standard procedures of PN supply as far as possible. This requires blinded IP in those sites where the patient will admix the PN including the lipid by themselves. The production of the blinded IP is at the sponsor site and the sponsor’s Qualified Person for IP is responsible for blinding the samples prior to shipment to the sites. In study sites where either the hospital pharmacy or a CU is involved in preparing the all-in-one PN admixtures including the IP as the lipid part, unblinded IP will be delivered to the pharmacy/CU. The PN including the lipid will be prepared and provided to the participating patient as in routine practice. Treatment allocation is realized by randomization envelopes, which are issued by the independent statistician and provided to an unblinded pharmacist (or a delegate). Of note, the blinding of the investigators or other staff outside the pharmacy/CU will be maintained throughout the complete process. Finally, the PN bag label of the pharmacy/CU will not reflect which lipid is used, but only the amount of lipid. Thus, it is guaranteed also that the patient is kept blinded.

Based on the randomization, list sets of emergency envelopes have been prepared for each participating site and the sponsor in case unblinding becomes necessary. Except for emergency reasons, the study will only be unblinded after closure of the database and determination of the analysis populations in a blinded data review meeting.

### Interventions and procedures

**Fig. 1:**
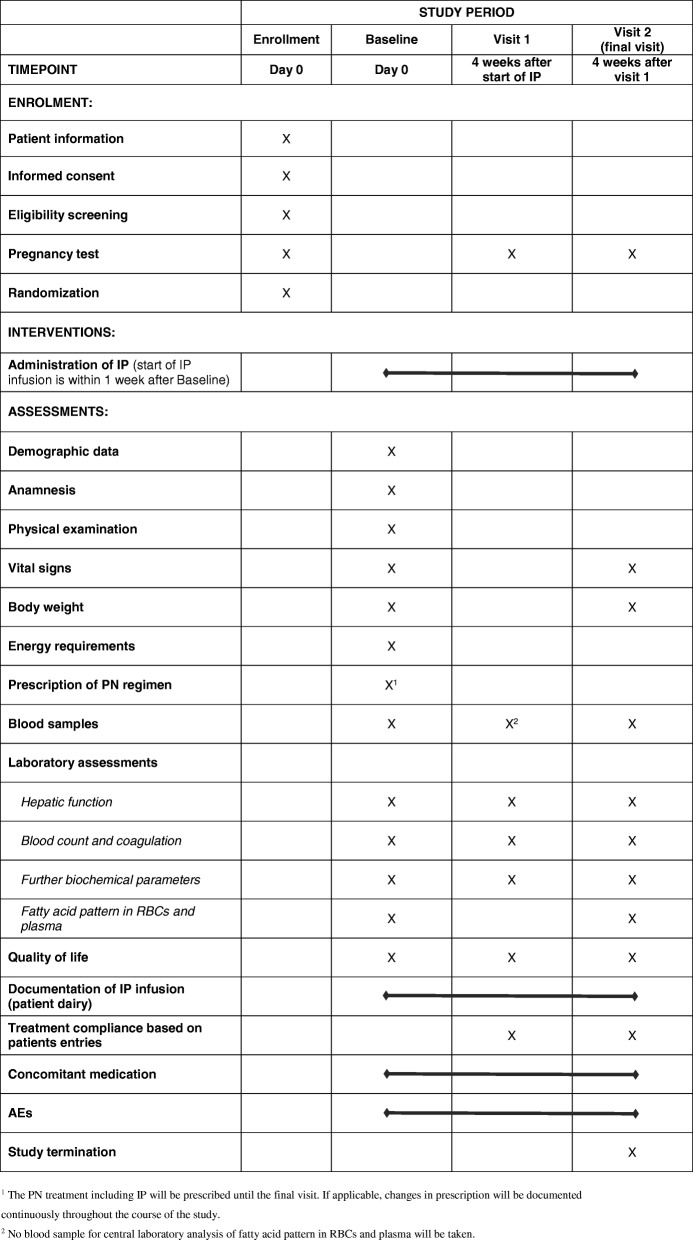
Standard Protocol Items: Recommendations for Interventional Trial (SPIRIT) Figure: HOME study visits. ^1^The parenteral nutrition (PN) treatment including Investigational Product (IP) will be prescribed until the final visit. If applicable, changes in prescription will be documented continuously throughout the course of the study. ^2^No blood samples for central laboratory analysis of fatty acid pattern in red blood cells and plasma will be taken

The schedule of enrollment, interventions and assessments is shown in Fig. 1 (SPIRIT Figure).

#### Screening and randomization

Patients will be enrolled for the trial from the existing patient pool of the participating clinic and information about the trial can be provided in advance by phone and with information materials. After obtaining informed consent, the patient will be checked for eligibility. If all inclusion criteria are met and all exclusion criteria are excluded the patient will be randomized.

#### Baseline visit

The baseline assessment is performed after randomization on the day of screening. Collected and generated data will be recorded in electronic Case Report Forms (eCRFs). Patient characteristics (demographic data, anamnesis and physical examination) and vital signs will be documented. Routine blood samples are taken and results for hepatic function, blood count and coagulation as well as biochemical parameters will be documented for the trial. For central laboratory analysis of the primary variable and the fatty acid (FA) pattern in red blood cells (RBCs) and plasma, additional blood samples are drawn and processed. Blood samples are taken no sooner than 3 h after completion of the last PN infusion. Patients will be asked to complete a quality of life (QoL) questionnaire (EQ-5D-5 L™, EuroQol Group, (Herdman et al., 2011)). Concomitant medication and adverse events (AEs) will be monitored and recorded continuously from study start (randomization) throughout the duration of patient’s study participation.

Patients are provided with a diary for documentation of IP administration on a daily base until visit 1. The patient has to enter date, start and end time of infusion of each PN bag containing the IP as well as the weight of the bag before and after infusion. Furthermore, the intake of oily fish meals is recorded.

The PN regimen including IP will be prescribed and prepared as needed. The infusion of IP will start within 1 week after baseline visit. If applicable, changes in prescription will be documented throughout the course of the study.

#### Visit 1

The study visit 1 will be conducted 4 weeks after start of IP infusions. The visit should be preferably the same day of the week and time of day as the baseline visit. If this is not possible, it should be the same interval between PN infusion and visit day as it was for baseline (e.g., if the baseline visit was after a day free of PN, visit 1 should also be after a day free of PN).

Depending on patient’s travel distance and organizational circumstances, the study visit can be scheduled around the intended visit date within a tolerance timeframe of − 3 days up to + 2 weeks.

All assessment of safety, efficacy and other variables will be performed except blood draw for FA pattern and the QoL questionnaire. The first part of the patient diary will be collected and checked for accurateness and completeness. The data will be entered into the eCRF and the second part of the patient diary will be handed out for the treatment period until visit 2.

#### Visit 2 (final visit)

Visit 2 will be conducted 4 weeks after visit 1 and is the final visit. As for visit 1 it should also be preferably the same day of the week and time of day as the baseline visit (and/or visit 1). If this is not possible, it should be the same interval between PN infusion and visit day as it was for visit 1. The tolerance timeframe for visit 2 is − 3 days up to + 2 weeks around the intended visit date. All assessment of safety, efficacy and other variables will be repeated at visit 2. The second part of the patient diary will be collected. After visit 2 the study ends for the patient and no IP must be administered. The PN treatment will continue according to the decision of the physician with a regularly used ILE.

### Study and treatment duration

The study starts with randomization and ends with the final visit (visit 2). The IP administration will start within 1 week after randomization and the duration of treatment with IP is projected to be a period of 8 weeks on average.

### Outcome measures

#### Primary outcome

The primary objective of the study is to prove safety and tolerability of HPN with an n-3 PUFA-enriched ILE in adult patients with CIF in need of long-term HPN. It aims to show non-inferiority of the test IP in comparison to the reference IP with regard to liver function.

The primary endpoint of the study is the change of liver function parameters defined as the sum of the *N* (0,1)-transformed differences in bilirubin, ALT and AST from baseline to visit 2:

$$ \Delta =\frac{\mathrm{BILI}-{\Delta}_1}{{\mathrm{SD}}_1}+\frac{\mathrm{ALT}-{\Delta}_2}{{\mathrm{SD}}_2}+\frac{\mathrm{AST}-{\Delta}_3}{{\mathrm{SD}}_3}, $$

where *BILI* = change of total bilirubin, *Δ*_*1*_ = mean change of *BILI*, *SD*_*1*_ = standard deviation of change of BILI; *ALT* = change of SGPT, *Δ*_*2*_ = mean change of ALT, *SD*_*2*_ = standard deviation of change of ALT; *AST* = change of SGOT, *Δ*_*3*_ = mean change of AST and *SD*_*3*_ = standard deviation of change of AST.

#### Secondary outcomes

The secondary objectives are the further evaluation of safety and efficacy. Secondary safety variables include parameters of hepatic function, blood count and coagulation, blood biochemistry, triene:tetraene ratio calculated from plasma FA pattern and AEs. Body mass index (BMI) and FA pattern in plasma and RBCs are secondary efficacy variables. Other secondary variables are demographic data, anamnesis and physical examination, vital signs, calculated BW change, QoL according to EuroQol Group EQ-5D™, concomitant medication, energy requirements, PN prescription per week and treatment compliance. Detailed secondary safety, efficacy and other variables are listed in Additional file 1.

### Concomitant medication, therapies

The following concomitant medications are allowed:

- Nutritional components (glucose, amino acids, vitamins, trace elements and electrolytes) according to the patient’s needs and investigator’s prescription either in a two-chamber bag or in a compounded all-in-one PN-bag to which the IP will be added
- Due to manufacturing/transportation steps there is a time interval between the baseline visit and the first administration of IP (maximum of 1 week). During this time-frame the administration of the usually prescribed lipid emulsion is allowed until the IP is provided to the patient
- Administration of concomitant medication as clinically required, but always considering the IP’s special warnings and precautions for use

The following concomitant medications are not allowed:

- With the exception of the situation as described above (maximum interval of 1 week after baseline visit), the administration of lipid emulsions other than IP
- Any dietary supplements containing n-3 PUFA including, but not limited to, fish-oil capsules, enteral/oral nutritional supplements containing fish-oil etc.

### Safety evaluation and reporting of adverse events

Throughout the course of the clinical trial, from the moment of randomization until the last study visit, particular attention will be paid to all AEs including serious AEs (SAEs). The investigator must record all AEs in detail whether serious or not. SAEs have to be reported to the sponsor within 24 h after the first knowledge that they have occurred.

SAEs with suspicion of a causal relationship to the study treatment (serious adverse reaction, SAR) that are unexpected according to the available summaries of product characteristics of Lipidem® 200 mg/ml and Lipofundin® MCT/LCT 20% have to be considered as Suspected Unexpected Serious Adverse Reactions (SUSARs). SUSARs are subject to expedited reporting. The sponsor will notify the competent authorities, Ethics Committees and all investigators concerned about SUSARs, in line with pertinent legal requirements.

The entire clinical study might be discontinued upon an unexpected high frequency of (S)AEs/(S)ARs or the occurrence of SUSARs. Individual patients might be withdrawn by the investigator in case of (S)AEs leading to non-acceptance of study continuation.

### Statistical methods

#### Sample size calculation

The sample size calculation is based on the primary endpoint of the study, which is the change of liver function parameters defined as the sum of the *N* (0,1)-transformed differences from baseline in bilirubin, ALT and AST after 8 weeks of treatment. It is the aim to demonstrate non-inferiority of the test IP as compared to the reference IP with respect to “deterioration in liver function.” Because of the normality and equal variance, the one-sided, two-sample *t* test will be applied. The standard deviation of the specified endpoint was derived from previous study data providing a standard deviation of *σ* = 2.3029, so the non-inferiority margin for this study is taken as *σ*/2, i.e., δ = 1.151. For the sample size calculation the power was taken as 1 − *β* = 0.8 and the significance set at *α* = 0.025, resulting in a sample size of 128 patients for two groups (64 per group). Assuming a drop-out rate of 20%, a total of 160 patients should be randomized in a ratio of 1:1 for assessment of the primary endpoint.

#### Analysis populations

The All-Patients-Screened population will comprise all patients who gave written informed consent to participate in the study. The Intention-To-Treat Set will comprise all randomized patients enrolled in the study. The All-Patients-Treated Set will comprise all patients of the ITT set who received at least one dose of the trial medication.

Additionally, the Full Analysis Set (FAS) will comprise all patients who received at least one dose of the trial medication and from whom at least one efficacy measurement is available after this dose. The Valid Case Analysis Set (VCAS) will comprise all patients in the FAS who did not show any major violations of the protocol including the violations of the requirements of study conduct.

#### Statistics

All programming of tables, figures, listings and statistical analyses will be performed using a statistical software package (SAS® version 9.4). Statistics will be performed in accordance with the principles outlined by the guideline E9 of the International Conference on Harmonization (ICH) and will be outlined in detail in the statistical analysis plan that will be finalized before close of database.

The primary analysis will be performed for the VCAS (FAS for sensitivity). No imputation will be performed. The *t* test will compare the (one-sided) null hypothesis (*H*_0_: *Δ*_T_ ≥ *Δ*_S_ + *δ*) against the alternative hypothesis (*H*_1_: *Δ*_T_ < *Δ*_S_ + *δ*) on the level of 0.025, where *Δ*_T_ and *Δ*_S_ represent the increase of the primary endpoint upon T = test and S = standard, respectively. Non-inferiority (with non-inferiority margin *σ* = 1.151) can be concluded, if the VCAS analysis yields an upper limit of the two-sided 95% confidence interval (or one-sided 97.5% confidence interval) for the treatment contrast of the primary endpoint lower than 1.151 and if this result is confirmed by the corresponding FAS analysis. Superiority of T vs. S, i.e., H0: *Δ*_T_ ≥ *Δ*_S_ vs. H1: *Δ*_T_ < *Δ*_S_, is demonstrated simultaneously if the upper limit of the confidence interval is below zero for both the FAS analysis and the VCAS analysis.

Given non-inferiority of T vs. S, breakdown of the multiple primary endpoint to its components bilirubin, ALT and AST, will be performed [28]. Further methods are descriptive statistics including standard procedures for the comparison of two groups (*t* test, Mann-Whitney *U* test, *χ*^2^ test).

Tests of secondary variables will be carried out in the area of exploratory data analysis. Therefore, corresponding *p* values are to be regarded as exploratory ones and no adjustments for multiple testing will be made.

### Data registration and monitoring

All data obtained in the context of the clinical trial are subject to data protection. If personal data are stored and processed, the requirements of pertinent data protection legislation will be observed.

Every effort will be made to collect all data points in the study. The amount of missing data will be minimized by appropriate management of the trial, proper screening of subjects, and training of participating investigators and other authorized staff, monitors and study manager.

The data generated in this study will be recorded using a computerized system in accordance with applicable regulations. The system will generate an individual eCRF for each patient participating in the trial. The principal investigator of each study site must ensure the accuracy, completeness and timelines of the data entry in the system. The eCRF system will guarantee compliance with Code of Federal Regulations (CFR) 21 part 11, data safety, communication security, limited access, user roles and full audit trail.

Authorized, qualified clinical research associates will visit the investigational sites at regular intervals as defined in the monitoring plan to verify adherence to the protocol and local legal requirements, to perform source-data verification and to assist the investigator in their study-related activities. Facilities that are involved in IP handling and dispensing will also be visited regularly for monitoring of IP accountability. In the case that unblinded IP is used at the facility, an additional unblinded monitor will perform these visits. An independent audit at the study site may take place at any time during or after the study.

### Ethical and legal considerations

This clinical study will be conducted in accordance with the ethical principles described in the Declaration of Helsinki and in compliance with the protocol, Good Clinical Practice (2001/20/EEC, CPMP/ICH/135/95), designated standard operating procedures, and with local legal and regulatory requirements in the countries in which the study will be conducted. Before the start of the trial, the trial protocol and all documents that are subject to review will be provided to the Ethics Committees/Institutional Review Boards concerned, and to competent national and local authorities by the sponsor or the investigator in line with national provisions. All substantial protocol modifications will be submitted as substantial amendments to these committees and competent authorities.

Informed consent has to be obtained from all patients. The patients will be advised that they have the right to withdraw from the study at any time without prejudice, and may be withdrawn at the investigator’s/sponsor’s discretion at any time, when this is considered to be in the interest of the patient.

### Responsibilities

Responsibilities of investigators, monitors and sponsor of the clinical trial regarding handling and storage of data, planning, assessment and quality assurance are regulated by the recommendations on “International Conference on Harmonization Topic E6 Guideline for Good Clinical Practice” and apply also to this clinical trial.

The costs necessary to perform the study have been agreed upon with each investigator and are documented in separate financial agreements which have been signed by the hospital administration, the investigator and the sponsor, prior to the study commencing.

The sponsor B. Braun Melsungen AG has taken out subject insurance for all patients taking part in the trial.

### Publication policy

The sponsor and principal investigators will agree on the final study report. It is intended that the results of the study may be published as scientific literature. In accordance with generally recognised principles of scientific collaboration, co-authorship with any sponsor personnel will be discussed before submission of a manuscript to a publisher. Results may also be used in submissions to regulatory authorities. Information developed in this clinical study may be disclosed as required to other investigators or any appropriate international regulatory authorities. The sponsor will be provided with complete test results and all data developed during this study.

### Study registration

The study protocol was registered in the ClinicalTrials.gov Protocol Registration and Results System, ClinicalTrials.gov, ID: NCT03282955 on 14 September 2017.

## Discussion

This prospective, randomized controlled clinical trial aims to prove the safety and tolerability of an n-3 PUFA-enriched ILE during HPN in adult patients with CIF and to show that the ILE containing n-3 PUFAs (test IP) is comparable to the ILE containing SO/LCT and MCT (1:1) with regard to liver function. Additionally, the study should provide further evaluation of safety and efficacy of the ILEs.

Only very few RCTs on the use of ILEs in adult CIF patients receiving HPN are published and, furthermore, the data on the use of n-3 PUFA and their influence on liver function are very limited. It was reported in a multicenter clinical trial that a FO containing ILE is safe and well-tolerated in CIF patients and leads to a decrease of liver values for total bilirubin, ALT and AST after 4 weeks [26]. A recently published RCT comparing four ILEs for 12 months showed that all tested ILEs were safe and comparable regarding their influence on liver function, but did not confirm a positive clinical impact of FO [27]. Other published reports on the use of FO/n-3 in adult CIF patients are only case reports or case series and, thus, there is a clear need for more data from randomized controlled trials.

The present study aims to include 160 stable adult CIF patients. This sample size is one of the biggest challenges in this study since CIF is an “orphan” disease and the rarest kind of organ failure. The prevalence of HPN for CIF due to benign disease has been estimated to be between 5 and 20/1,000,000 [3] and 3.25–66/1,000,000 [29] in Europe. A multicenter, multinational approach was chosen to accomplish the statistically required samples size, and also to increase the external validity and the international generalizability of the results.

The supply of PN for patients at home is subject to thorough organized processes and in this respect there are many differences between countries and even between hospitals within the same country. In this multicenter, multinational clinical trial it is intended to follow the individual standard procedure of PN preparation and supply as far as possible. For HPN patients in the United Kingdom (UK) several pharmacy service companies are available, but for this clinical trial only one compounding company was selected. This can be a limiting factor for recruitment in the UK, and also requires consideration when interpreting the data, in particular with regard to the generalizability of the results for the UK sites.

The primary endpoint is the combined change of the liver function parameters bilirubin, ALT and AST. Literature reporting on the incidence of liver disease in adults with CIF receiving long-term HPN varies in the biochemical and/or histological parameters used to define liver dysfunction [15, 26, 30]. A consensus definition, that sets parameters for the truly standardized diagnosis of IFALD is not available. We consider the combined change of bilirubin, AST and ALT useful and sensitive enough to detect and evaluate liver dysfunction. In order to complete the standard liver panel, alkaline phosphatase (ALP) and gamma-glutamyl transpeptidase (GGT) are assessed as secondary endpoints.

Most study assessments are part of the routine monitoring practice for long-term HPN patients. Normally, patients receiving HPN visit the outpatient clinic every 2–4 months. The intervals between the study visits are shorter and additional blood samples are drawn to analyze the primary endpoint centrally and for assessment of the secondary parameters including FA patterns. The questionnaire used for the subjective patient-reported QoL (EQ-5D-5 L) is well-accepted. The diary for documentation of investigational lipid infusion is designed in a way that the required information can be recorded with minimal effort. The prescription of the investigational lipid during the study is according to the prescription of the lipid before inclusion of the patient into the study. Both IPs have market authorization and have been used for years in different patient groups. Given that the patients participating in the study are dependent on nutritional treatment and the supply of lipids, the risks associated with the IP administration are assumed not to be higher than those when lipids are administered in their regular treatment. Taking together all these aspects, the burden and risks for patients are expected to be very low and the study protocol has limited impact on patients’ daily lives.

In summary, the HOME study should provide data from a considerable number of adult patients with CIF receiving HPN and thus contribute to broaden the evidence on the use of ILEs in HPN.

## Trial status

Currently, the trial is in the recruitment phase. Recruitment started on 8 January 2018 and the estimated completion date will be in July 2021. The study protocol has the current version 2.2, dated 5 November 2018. The change history is given in the “Ethical approvals” section.

## Supplementary information

**Additional file 1.** Secondary study variables.

**Additional file 2.** Standard Protocol Items: Recommendations for Interventional Trials (SPIRIT) Checklist.

## Data Availability

Not applicable

## Abbreviations

AE: Adverse event
ALP: Alkaline phosphatase
ALT: Alanine transaminase
AST: Aspartate transaminase
BMI: Body mass index
BW: Body weight
CIF: Chronic intestinal failure
CU: Compounding unit
DHA: Docosahexaenoic acid
eCRF: Electronic Case Report Form
EFA: Essential fatty acids
EPA: Eicosapentaenoic acid
ESPEN: The European Society for Clinical Nutrition and Metabolism
FA: Fatty acid
FAS: Full Analysis Set
FO: Fish oil
GGT: Gamma-glutamyl transpeptidase
HPN: Home parenteral nutrition
IFALD: Intestinal-failure-associated liver disease
ILE: Intravenous lipid emulsion
IP: Investigational Product
IVS: Intravenously administered supplementation
LA: Linoleic acid
LCT: Long-chain triglyceride
MCT: Medium-chain triglyceride
n-3: Omega-3
n-6: Omega-6
OO: Olive oil
PN: Parenteral nutrition
PUFA: Polyunsaturated fatty acid
QoL: Quality of life
RBC: Red blood cell
RCT: Randomized controlled trial
SAE: Serious adverse event
SAR: Serious adverse reaction
SD: Standard deviation
SO: Soybean oil
SPIRIT: Standard Protocol Items: Recommendations for Interventional Trials
SUSAR: Suspected Unexpected Serious Adverse Reaction
VCAS: Valid Case Analysis Set
