# Supplementary material for: Home parenteral nutrition with an omega-3-fatty-acid-enriched MCT/LCT lipid emulsion in patients with chronic intestinal failure (the HOME study): study protocol for a randomized, controlled, multicenter, international clinical trial
Source: Trials. 2019 Dec 30;20:808. doi: 10.1186/s13063-019-3994-z (PMC6938010; doi:10.1186/s13063-019-3994-z)
Supplement: Supplementary file 1 — Additional file 1. Secondary study variables. [file 13063_2019_3994_MOESM1_ESM.docx]

### Additional file 1: Secondary study variables

Variables indicated with * are calculated values

**Safety**

- Hepatic function
- Bilirubin (total and conjugated)
- Alanine transaminase (ALT)
- Aspartate transaminase (AST)
- AST/ALT ratio*
- Alkaline phosphatase
- Gamma-glutamyl transpeptidase
- Blood count and coagulation
- White blood cells
- Red blood cells
- Hemoglobin
- Hematocrit
- Platelets
- International normalized ratio (if not possible prothrombin time [prothrombin time = Quick-value] is accepted)
- Activated partial thromboplastin time
- Other biochemical parameters
- Blood glucose
- Electrolytes (Na, Cl, K, Ca, Mg, P)
- Serum creatinin
- Triglycerides
- Cholesterol
- High-density lipoprotein
- Low-density lipoprotein
- C-reactive protein
- α-Tocopherol/Vitamin E (facultative if routinely assessed)
- Triene:tetraene ratio* obtained from fatty acid pattern in plasma
- Adverse events

**Efficacy**

- Fatty acid pattern in plasma and RBCs
- BMI*

**Other variables**

- Demographic data
- Age
- Gender
- Ethnic origin
- Body height
- Body weight
- Anamnesis
- Medical history relevant with regard to HPN
  - Pathological classification of Intestinal Failure
  - Underlying disease
- Concomitant disease(s)
- Ongoing medications
- Lipid emulsion(s) during the last 6 months
- Anamnestic peculiarities
- Physical examination
- Vital signs
- Body weight change*
- Quality of life (EQ-5D^TM^)
- Concomitant medication
- Energy requirements
- PN regimen prescription
- Treatment compliance*
- Intake of oily fish meals

### Study termination
